# Repetitive mild TBI causes pTau aggregation in nigra without altering preexisting fibril induced Parkinson’s-like pathology burden

**DOI:** 10.1186/s40478-022-01475-9

**Published:** 2022-11-26

**Authors:** Vedad Delic, Joshua H. Karp, Maynard Guzman, Gabriel R. Arismendi, Katherine J. Stalnaker, Julia A. Burton, Kathleen E. Murray, Joshua P. Stamos, Kevin D. Beck, Arpine Sokratian, Andrew B. West, Bruce A. Citron

**Affiliations:** 1 Laboratory of Molecular Biology, VA New Jersey Health Care System, Research and Development (Mailstop 15), Bldg. 16, Rm. 16-130, 385 Tremont Ave, East Orange, NJ 07018 USA; 2 Neuro Behavioral Research Laboratory, VA New Jersey Health Care System, Research and Development (Mailstop 15), Bldg. 16, Rm. 16-130, 385 Tremont Ave, East Orange, NJ 07018 USA; 3 Neurology Service, VA New Jersey Health Care System, 385 Tremont Ave, East Orange, NJ 07018 USA; 4 Department of Pharmacology, Physiology, and Neuroscience, Rutgers- New Jersey Medical School, Newark, NJ 07103 USA; 5 Department of Neurology, Rutgers- New Jersey Medical School, Newark, NJ 07103 USA; 6 Rutgers School of Graduate Studies, Newark, NJ 07103 USA; 7 Neurobiology Department, Department of Pharmacology and Cancer Biology, Duke Center for Neurodegeneration Research, Duke University School of Medicine, Durham, NC 27710 USA; 8 Duke University School of Medicine, Durham, NC 27710 USA

## Abstract

Population studies have shown that traumatic brain injury (TBI) is associated with an increased risk for Parkinson’s disease (PD) and among U.S. Veterans with a history of TBI this risk is 56% higher. The most common type of TBI is mild (mTBI) and often occurs repeatedly among athletes, military personnel, and victims of domestic violence. PD is classically characterized by deficits in fine motor movement control resulting from progressive neurodegeneration of dopaminergic neurons in the substantia nigra pars compacta (SNpc) midbrain region. This neurodegeneration is preceded by the predictable spread of characteristic alpha synuclein (αSyn) protein inclusions. Whether repetitive mTBI (r-mTBI) can nucleate PD pathology or accelerate prodromal PD pathology remains unknown. To answer this question, an injury device was constructed to deliver a surgery-free r-mTBI to rats and human-like PD pathology was induced by intracranial injection of recombinant αSyn preformed fibrils. At the 3-month endpoint, the r-mTBI caused encephalomalacia throughout the brain reminiscent of neuroimaging findings in patients with a history of mTBI, accompanied by astrocyte expansion and microglial activation. The pathology associated most closely with PD, which includes dopaminergic neurodegeneration in the SNpc and Lewy body-like αSyn inclusion burden in the surviving neurons, was not produced de novo by r-mTBI nor was the fibril induced preexisting pathology accelerated. r-mTBI did however cause aggregation of phosphorylated Tau (pTau) protein in nigra of rats with and without preexisting PD-like pathology. pTau aggregation was also found to colocalize with PFF induced αSyn pathology without r-mTBI. These findings suggest that r-mTBI induced pTau aggregate deposition in dopaminergic neurons may create an environment conducive to αSyn pathology nucleation and may add to preexisting proteinaceous aggregate burden.

**Supplementary Information:**

The online version contains supplementary material available at 10.1186/s40478-022-01475-9.

## Introduction

Idiopathic Parkinson’s disease (PD) is the most common neurodegenerative movement disorder affecting millions of people. Death of A9 dopaminergic neurons (DN) causes postural instability, resting tremors, and freezing gate that together lead to a PD diagnosis most frequently in the 6th decade of life [1, 2]. DN loss is preceded by nonrandom spread of characteristic intracellular alpha synuclein inclusion pathology through interconnected brain areas, caudally from the olfactory bulb or rostrally from the brainstem through substantia nigra pars compacta (SNpc) [3]. These inclusions, termed Lewy bodies (LB) and Lewy neurites (LN), are made up of various organellar fragments and hyper phosphorylated Alpha synuclein (αSyn) protein. αSyn is an intrinsically disordered protein enriched in the nucleus and at the synapse that exists as a monomer in equilibrium with other oligomeric forms and in PD αSyn assumes an aggregation prone amyloid beta pleated sheet conformation [4]. PD inclusions have characteristic morphology, appearing as skein shaped perinuclear LBs or corkscrew LNs, and both are post translationally modified most notably by hyper-phosphorylation at serine-129 residue [5–7].

There are now 28 genes linked to hereditary late onset Parkinson’s disease and coinheritance of multiple of these variants further increase the risk for PD [8]. Due to pathology that is indistinguishable from idiopathic PD, some of these late onset variants are also implicated as potential contributors to idiopathic PD and continue to be under intense investigation [9]. Non genetic risk factors for PD include chemical exposures and traumatic brain injury (TBI). Exposures to certain herbicides and pesticides are known to cause selective death of dopaminergic neurons while exposure to industrial chemical pollutants e.g., trichloroethylene, in ground water is associated with an increased risk for PD [10, 11]. Millions of TBIs are reported each year making TBI the biggest non genetic risk factor for PD. Studies published between 2015 and 2020 indicate that the global incidence of TBI ranges from 476 per 100,000 in South Korea [12] to 787 per 100,000 in the USA [13]. High instances of sub-concussive repetitive mild TBI (r-mTBI) are common among military personnel, student athletes, and victims of domestic violence [14]. Patients with mTBI have good prognosis, even if immediately after injury small lesions are detected by neuroimaging [15]. TBI pathology is not static and even in the absence of clinical findings, pathology can evolve over days and weeks to include regional encephalomalacia caused by neuronal cell death, ventricular enlargement, astrocyte expansion and microgliosis [16]. In cases of mTBI these findings occur in the absence of motor deficits. Prospective and retrospective human population studies have shown that there exists a strong correlation between history of TBI and development of PD pathology [17–20]. U.S. Veterans with a history of TBI have a 56% higher risk for developing PD later in life, and this risk increases with severity of injury [14]. However, biological cause-and-effect evidence for de novo nucleation of authentic PD-like pathology or evidence for acceleration of existing PD pathology by TBI has not yet been established [21].

The goal of this study was to answer this fundamental question; *does r-mTBI cause or accelerate authentic human PD-like pathology in the SNpc?* We also sought to determine if the aggregation of hyper phosphorylated Tau (pTau) pathology, closely associated with TBI and reported elsewhere in the brain [22], also occurs in the SNpc. These experiments were accomplished by combining a well-established PD model initiated by SNpc injection of preformed αSyn fibrils (PFF) [23–26] with a novel rat model of repetitive diffuse mild TBI. The rat TBI model was adopted from previous work by Marmarou et al. [27] and the more recent work of Zohar et al. [28] and Buchele et al. [29]. This r-mTBI model produced encephalomalacia, reminiscent of pathology seen on neuroimaging of patients with history of TBI without causing motor or behavioral deficits [30]. We report histopathological analysis of our experiments at a timepoint previously shown using the PFF PD model to have robust αSyn pathology along with dopaminergic neurodegeneration [31]. Data herein shows that r-mTBI causes aggregation of pTau in the rat SNpc without causing de novo or accelerating preexisting αSyn pathology.

## Methods

### Animal usage

Male Sprague Dawley (SD) rats, 9–10 weeks of age, were procured from Charles River and were handled in accordance with the VA New Jersey Health Care System Institutional Animal Care and Use Committee (IACUC). Rats were housed individually in standard polycarbonate 18 quarts tubs with bed-o-cob bedding which was changed once a week. Individual housing was necessary due to planned behavioral experiments, and the unknown effect of r-mTBI together with PFF on the interaction between injured males in the same cage. Rooms were kept at 22 °C +/− 4 °C and rats were kept 12 h on and 12 h off reverse light cycles to allow for behavioral experiments during rat subject active phase. To minimize stress during handling and behavioral experiments rats were acclimated to the experimenter by handling for a week before experiments were performed. All behavioral experiments were performed under red light. Three separate cohorts were used in this study. Cohort 1: 4× r-mTBI and shams for behavior and histology, cohort 2: 8× r-mTBI and shams for behavior and histology, cohort 3: nucleation of PD by injection of PFFs followed by 8× r-mTBI for histology.

### Recombinant αSyn PFF synthesis

PFFs were generated in the lab of Dr. Andrew B. West at Duke University School of Medicine as previously described by Abdelmotilib et al. [26], with purification and quality control performed using dynamic light scattering (Fig. 5S). Briefly, murine αSyn was expressed in *E. Coli*, and produced in large batches. Crude protein extraction was achieved followed by size exclusion chromatography and ion exchange. Endotoxin was also removed. Purified αSyn was then aggregated into higher order fibrils using an aggregation assay by shaking at 37 °C for a week in buffer [24, 32]. These fibrils were fractured in a sonicating water bath, diluted to 5 µg/ml, and injected in a 4 µl volume over 30 min for total of 20 µg per injection site.

### Intracranial injection of monomeric αSyn, PFFs, or LPS

Rats were injected with monomeric αSyn to serve both as a vehicle and surgery control. PFF injection was used to corrupt the endogenous αSyn and promote human PD-like inclusion formation and pathology. Lipopolysaccharide (LPS) injection was used to validate antibodies and detection of astroglia expansion and microgliosis. Rats were anesthetized with 5% v/v isoflurane with a flowrate of 0.8 L/min. Once fully anesthetized, the rat fur was clipped, and the incision area cleaned with an alcohol wipe, and eye ointment was also applied. The rat was then placed on a heating pad in prone position, secured in the stereotaxic surgical system with ear bars, and kept under with a canulae delivering continuous O_2_ flow at 0.8 L/min and isoflurane setting of 2.5% v/v. Prior to incision the surgical area was further cleaned with chlorohexidine. For analgesia, subdermal injection of 0.1 ml Marcaine at 2.25 mg/kg was injected at each end of the planned sagittal incision which was made down the midline from approximately the frontal bone, moving caudally to the base of the skull to expose the entire calvarium. Once the incision has been made, the wound area was irrigated with sterile saline, and fascia attached to the skull was gently removed with sterile cotton applicators. To facilitate exposure of bregma, 3% hydrogen peroxide solution was applied directly to the skull. Once the bregma was clearly visible, a burr hole was made at the coordinates: X = 2.5, Y = 5.35–5.5, Z = 7.4–7.5 mm of Bregma. 4 µl volume of a 5 µg/µl PFF solution, monomeric alpha synuclein, or Lipopolysaccharide (LPS) was injected over 30 min. The needle was left in the injection site for 5 min after dispensing to prevent reflux along the injection track. The needle was retracted very slowly. Contralateral (left) side was intentionally left un-injected to serve as an internal control. After injections were complete, the surgical area of the exposed calvarium was irrigated with sterile saline, and the incision was closed with nylon surgical sutures. The sutured area was cleaned once more with 3% hydrogen peroxide and the rat was placed in a recovery cage with food and water on a heated pad. The rat was monitored for seizures and signs of recovery. After the initial 1-h observation during recovery from anesthesia the animal was placed back in the home cage, monitored once every hour, and then once every four hours.

### Traumatic brain injury

**Fig. 1:**
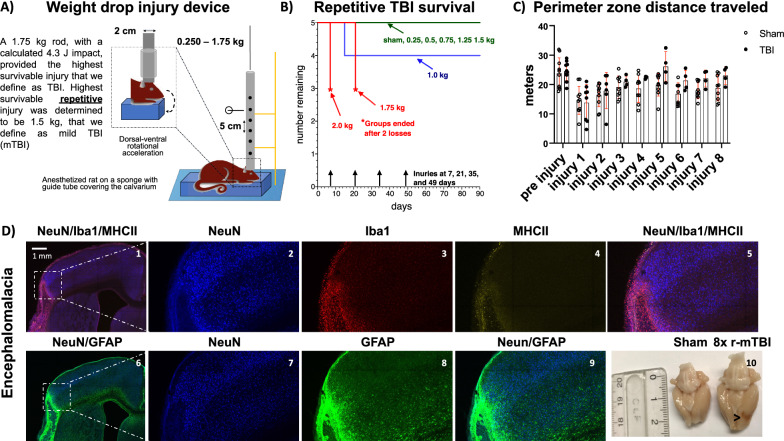
Gross pathology indicating encephalomalacia in rat brain following 8× rmTBI. Novel surgery-free, unshielded weight drop injury device designed to impact the entire calvarium similar to a fall, car accident, or strike to the head (**A**). Titration of injury severity 0–2.0 kg with 0.250 kg increments indicating instant fatality following a single 2.0 kg injury, instant fatality following second injury with 1.75 kg and survivability of 4 injuries with 1.5 kg weight drop injury from 25 cm height. **B** Behavioral assessment of motor deficits using an open field test 10 days post injury indicating no behavioral deficit after up to 8× r-mTBI. **C** Fluorescent staining for NeuN+, Iba1, MHCII, GFAP of an area of encephalomalacia (**D**_**1**_–**D**_**10**_). NeuN+ cells are absent from the area of encephalomalacia (**D**_**1**_–**D**_**2**_). The area of injury devoid of neurons (NeunN+) is infiltrated by Iba1+ and MHCII + microglia (**D**_**2**_–**D**_**4**_). Expansion of GFAP+ astrocytes in the area of injury (**D**_**10**_). An area of brain thinning or encephalomalacia is present and visible on the anatomical left cortex on an intact brain prior to sectioning. Dashed white line and square indicate region that was magnified in **D**_**2**_–**D**_**5**_ and **D**_**7**_–**D**_**9**_

The injury device was designed to approximate the most common type of head injuries which involve an impact to a large part of the skull from a fall, strike to the head, or contact with a hard surface during a vehicle collision. The injuries were performed once every 14 days to accommodate behavioral tests and every 10 days starting at 1 month after injection of PFFs and/or monomeric αSyn. Fully anesthetized male SD rats, 8–10 weeks of age, were placed on ∼10 cm, 2.84 N/cm stiffness foam platform in a prone position to allow for dorsoventral and anteroposterior acceleration and deceleration of the head [29] under a 2.5 cm diameter suspended guide tube covering the unshielded calvarium (Fig. 1A). Stainless steel injury weights ranging from 0.250 to 2.0 kg with 0.250 kg increments were loaded 25 cm above the head into the guide tube and held in place with a pin. The injury was initiated by rapidly pulling the pin allowing the weights to free fall 25 cm down a guide tube impacting the head. The weights were immediately retracted up the guide tube using an attached string. Injuries were titrated and TBI was defined as the highest survivable single injury (1.750 kg released from 25 cm and calculated impact of 4.29 J) within a 10–14-day period, not resulting in skull fracture or damage to the skin. We define mTBI (1.500 kg released from 25 cm with calculated impact of 3.68 J) as the highest survivable repetitive injury.

### Open field test for assessment of ambulatory activity

On day 1, starting with the open field, behavioral tests were performed on rats that underwent 8× r-mTBIs and cohort sham controls in the second week after each of the 8 injuries to measure long lasting consequences of r-mTBI. On day 1, rats were placed individually in a 72.4 cm by 72.4 cm square open arena, for 5 min with the center zone set 10 cm away from the edge of the arena. Area of the center zone is equal to that of the perimeter zone (area between wall of the arena and perimeter of the center zone). ANY-maze software version 6.17 was used to track the rat speed and location through a ceiling mounted camera.

### Novel object recognition and novel object placement memory tests

On day 2 of behavior, two identical objects were placed equidistant from each other, and the walls of the arena allowing animals to acclimate and explore the objects for 5 min. On day three (memory), two new objects were placed inside the arena; the rat was placed inside along the wall at the start of the test and allowed 5 min to explore. The animals were then placed back on the cart and remained in the test room for four hours after which one of the objects was replaced with a new object to test for novel object recognition. Position of the new object was alternated between left and right location to account for potential area rather than novel object preference. Again, animal position was tracked, and time spent exploring objects was recorded using Anymaze 6.17 software. On day four, similar to day 3, animals were placed into the arena with two new objects for five minutes (learning), and after a four-hour delay one of the objects was moved to a new location. Time spent exploring the objects at the original and new location was measured. Initial object selection criteria were made on the basis that the object cannot be moved by the rat, cannot be climbed on, can be easily cleaned, does not emit an odor, and had a variable textured surface. All objects were calibrated with non-experimental naïve rats prior to use to avoid aversion or preference to a specific object rather than a novel object. Novel objects that were preferred by naïve animals, or toward which the animal showed aversion were excluded from use in the experiments. Objects used included the following types: soda can, glass vase, various plastic soda bottles, jars, and cups.

### Perfusions and brain collection

Anesthesia was induced with 5% v/v isoflurane with a flowrate of 1.5 L/min. Once fully anesthetized, the rat fur was clipped, and the incision area cleaned with an alcohol wipe. The rat was then placed on a dissecting tray on its back, secured in place using pins, and kept anesthetized with a canulae delivering continuous O_2_ flow at 1.5 L/min and isoflurane setting of 5.0% v/v isoflurane. To ensure that the animal was fully anesthetized, hind limb pinch was performed to check for reflex. An incision was made, either on the right or the left lower quadrant below the rib cage, by cutting into the abdominal cavity. The incision was expanded along the one side by cutting the rib cage up to the clavicle, followed by an identical contralateral incision. The ribcage was then lifted and reflected, and the diaphragm was severed. The right atrium was cut for exsanguination, and a needle was inserted into the left ventricle, to first deliver 100 ml of PBS, followed by 100 ml of 4% PFA for fixation. The skull was opened using bone rongeurs. The exposed brain was removed gently using a small spatula and transferred to a 50 ml tube, with 4% PFA in PBS on a tumbler at 4 °C overnight followed by 48 h in 30% sucrose for cryopreservation. Lastly, the brain was rapidly frozen by submerging the cryoprotected brain for 2 min into methyl butane cooled with dry ice to − 40 to − 50 °C and stored at − 80 °C.

### Immunohistochemistry

40-micron serial coronal sections of the frozen cortex and midbrain were obtained using a freezing microtome. A needle borehole was used to indicate the side contralateral to the injection of fibrilized or monomeric αSyn. SNpc containing brain regions were sampled every 4th section and the rest of the midbrain including striatum was sampled every 6th section. Sections were stored at − 20 °C in 50% glycerol with 0.1% sodium azide. Entire nigral and striatal representative samples to be stained were removed from cryopreservation media and washed in TBS. Sections were then exposed to antigen retrieval solution (10 mM sodium citrate, 0.05% Tx-100, pH 6.0). Antigen retrieval solution was washed from the free-floating section with TBS before blocking in 5% goat or donkey serum (depending on the secondary antibody species) and 0.3% Tx-100 in TBS. Sections were then incubated in primary antibody for 24 h and secondary antibody solutions for 2 h at 4 °C with agitation (Table 1). Once mounted on a slide the sections were counterstained with Hoechst 33342 for nuclei. Sections for confocal microscopy were wet mounted using Pro-Long Gold and covered before imaging. LI-COR IRDye secondary antibodies were used for visualization using the LI-COR Odyssey scanner (Table 1).

**Table 1:** Primary and secondary antibodies used in the study

	Species	Type	Dilution	Vendor	Catalog #	Lot #
Primary antibody						
TH	Chicken	Polyclonal	1–1000	Millipore	AB9702	3519393
NeuN	Mouse	Monoclonal	1–1000	Novus	NBP1-92693	D102247
IBA1	Rabbit	Polyclonal	1–1000	Wako	019-19741	CAE1308
MHC II	Mouse	Monoclonal	1–500	BD	BDB554926	1137896
GFAP	Chicken	Polyclonal	1–1000	EnCor	CPCA-GFAP	7529-5
pSyn	Rabbit	Monoclonal	1–1000	Abcam	ab51253	GR3232346-6
pTau (AT8)	Mouse	Monoclonal	1–2000	ThermoFisher	MN1020	WJ3381064
s100Beta	Rabbit	Monoclonal	1–1000	Abcam	Ab54642	GR3215095-29
s100Beta	Rabbit	Monoclonal	1–1000	Novus	NBP2-53188	6285-4P10803
s100Beta	Mouse	Monoclonal	1–1000	ProteinTech	6616-Ig	10004814
Secondary antibody						
Alexa 488 anti-rabbit	Goat	Polyclonal	1–1000	Invitrogen	A32721	UD282059
Alexa 555 anti-chicken	Goat	Polyclonal	1–1000	Invitrogen	A21437	2352144
Alexa 647 anti-mouse	Goat	Polyclonal	1–1000	Invitrogen	A32728	UD279293
IRDye 680RD anti-rabbit	Donkey	Polyclonal	1–10,000	LICOR	925-68073	C90129-11
IRDye 800CW anti-mouse	Donkey	Polyclonal	1–10,000	LICOR	926-32212	D20126-04
HRP anti-rabbit	Goat	Polyclonal	1–1000	Cell signaling	7074P2	31
HRP anti-mouse	Goat	Polyclonal	1–2000	Invitrogen	62-6520	UA281872

The antibodies were internally validated, and optimal concentrations determined

### Fluorescent confocal microscopy and imaging

Sections were imaged in four channels using Olympus FluoView 3000 confocal microscope and Fluoview FV31S-SW software. Excitation wavelengths of 405 nm, 488 nm, 561 nm, and 640 nm were optimized in each channel before images were acquired. For counting TH, pSyn, and pTau, 10× magnification z-stack images (10 µm step size) of ipsilateral and contralateral sides were obtained. Each side was stitched together separately using the FV31S-SW software. Z-stack images for visualization of pSyn, pTau, and inflammatory marker pathology were obtained at 20× and 60× magnification using the same optimized laser settings. All images were exported and processed to MaxZ projections using ImageJ 1.53a [33].

### *Counting TH* + *neurons, Lewy body-like inclusions and pTau aggregates*

PD related pathology was counted in the SNpc containing brain slices sampled every 4th section. 10× magnification z-stack images of SN stained for tyrosine hydroxylase (TH), phosphor-Serine-129 alpha synuclein (pSyn), and phosphorylated Tau (pTau) were processed in ImageJ to MaxZ projections. TH stain locality was used to identify and draw a region of interest (ROI) around the SNpc in each image. Files with drawn ROI were randomized using a better finder rename 10 software. The files were then assigned to be counted by a technician blinded to treatment groups and brain injection location for unbiased counting of dopaminergic neurons (TH+), Lewy bodies (pSyn+), and pTau+ inclusions. DNs were counted only if an entire cell body was within the drawn ROI that had TH+ staining. Axonal projections and non-spherical/partial cell bodies were not counted. Lewy body-like inclusions were counted with stringent exclusion criteria. Initial counts of Lewy bodies were obtained by identifying pSyn+ aggregates with stain intensity greater than 3× background intensity and co-localized to a TH+ neuron body. The pSyn+ inclusions had to be perinuclear and within the vicinity of other Lewy Bodies or Lewy neurites in the same or adjacent TH+ neuron. Images that met some but not all exclusion criteria were flagged and imaged at higher magnification. pTau inclusions were counted with several stringent exclusion criteria. A pTau+ aggregate was counted as an inclusion only if the pTau+ aggregate co-localized to a TH+ cell body and cell-like morphology was apparent. Punctate staining, i.e. 1–2 pixels of increased stain intensity, were not counted as a pTau inclusion. pTau inclusions were counted as total pTau inclusions per image as well as pTau inclusions that co-localized with pSyn+ Lewy bodies. Using the criteria each object was selected using multi point tool in imageJ, to click and record each counted object. In each case, the counts were normalized to the area of the SNpc reported in mm^2^. Samples of counting and selections are included in the supplemental section (Fig. 6S).

### LICOR imaging

A LI-COR Odyssey CLx scanner was used to visualize gross brain pathology on coronal sections from 8× r-mTBI rat brains. Sections mounted on slides were scanned tissue side down without a coverslip on the scanner bed, with an offset of 0.00 mm and a 21 μm resolution. The area of capture was set using LI-COR software. Double staining with antibodies against Iba1 and MCHII (Table 1) were used to distinguish areas of authentic pathology from potential tissue damage during cutting and mounting. George Paxinos and Charles Watson SCR_017124 Rat Brain Atlas was used for reference [34].

### s100Beta measure in blood for TBI ELISA and western

s100Beta, an astrocytic protein, has been reported as a peripheral blood biomarker of TBI severity in humans and rats [35]. The greatest blood brain barrier (BBB) breach was reported 2 h after a TBI in rats [36]. To determine the degree of BBB permeability caused by our TBI and mTBI, blood samples were collected from control and TBI rats 2 h after a closed head weight drop injury. Deeply anesthetized rats underwent a bilateral thoracotomy followed by a cardiac puncture for blood collection. Serum was separated out by centrifugation. Serum sample and quality controls were prepared per s100beta kit (BioVendor R&D^®^). instructions. Absorbance was measured using a plate reader at 450 nm, with the reference wavelength set to 630 nm.

For western blot analysis, 30 μg of serum protein was loaded on a 4–20% stain free Mini-Protein TGX Gel (Bio-Rad) for 30–40 min at 200 V. The gel was transferred to a methanol activated PVDF membrane, using Trans-Blot Turbo mini packs (Bio-Rad) on a Trans-Blot Turbo ™ System. After 7-min transfer, the membrane was washed and blocked using 5% blocking buffer in TBST. The membrane was probed with 3 most frequently cited and commercially available s100beta antibodies with corresponding HRP conjugated secondary (Table 1). Membranes were incubated with primary antibody in 5% blocking solution with agitation overnight at 4 °C. After washing, the membranes were then incubated for 2 h at 4 °C with appropriate HRP conjugated secondary and visualized using ChemiDoc MP imaging system (Bio-Rad). The signal was normalized to total protein on the membrane. Briefly, stain free gels allow for cross linking of loaded protein using UV light before transfer. After transfer UV is used again on the membrane, which allows for visualization of total protein bands on the membrane. All band quantification was performed using Image Lab studio for MacOS Version 6.1.0 build 7 2020. Dual Color Standard protein ladder was used for band size estimation. All transfer buffers used were obtained and used as indicated by the manufacturer’s instructions (Bio-Rad).

### Statistics

Required sample size was determined experimentally, starting with n = 6 per group and increasing to n = 10 per group after initial fatalities and noted variability with behavioral experiments. For TH+ neurons zero values were not expected, ordinary one-way ANOVA was performed to test for significance with Holm-Šídák’s multiple comparisons test comparing all means to 8× r-mTBI+ monomer group. In counts of αSyn pathology where zero values were expected, the Kruskal–Wallis test was performed. Grubbs outlier tests were performed with alpha 0.01. In all groups where outliers were identified and removed from comparison, outlier values are included in the figure caption. Significance was indicated by *p < 0.05 and **p < 0.01. Open bar graphs indicate means, closed circles represent individual data points, and error bars represent standard error of the mean. Data was analyzed and graphed using Prism 9.3.1 for macOS.

## Results

### r-mTBI caused encephalomalacia without motor deficits

**Fig. 2:**
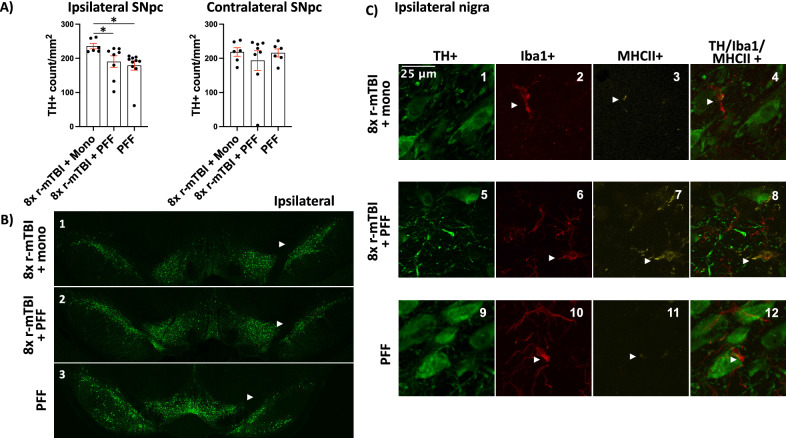
Dopaminergic degeneration in the SNpc 3 months after injection of PFFs, monomeric αSyn, and 8× r-mTBIs. Quantification of TH+ dopaminergic neurons in the SNpc was performed using imageJ and the counts were normalized to the area of the SNpc indicating a loss of neurons following injection of PFFs. PFF induced loss of neurons was not accelerated by 8× r-mTBIs (**A**). TH+ neuronal staining of the whole nigra from 8× r-mTBI + monomer, 8× r-mTBI + PFF, and PFF only rat brains indicating ipsilateral neuron loss in the 8× r-mTBI + PFF and in PFF only groups (**B**_**1**_–**B**_**3**_). White arrowhead indicates intact ipsilateral nigra (**B**_**1**_). Arrowhead indicates degenerating ipsilateral nigra (**B**_**2**_–**B**_**3**_). High magnification of nigra from 8× r-mTBI + monomer, 8× r-mTBI + PFF, and PFF only rat brains stained for TH, Iba1 and MHCII (**C**_**1**_–**C**_**12**_). MHCII+ iba1+ amoeboid microglia present in nigra of 8× r-mTBI + PFF rat brains (**C**_**6**_–**C**_**8**_). Low MHCII + staining in 8× r-mTBI + monomer (**C**_**2**_–**C**_**4**_). Low MHCII staining in PFF only group (**C**_**10**_–**C**_**12**_). White arrowheads indicate ramified microglia colocalizing with low MHCII signal (**C**_**2**_–**C**_**4**_). Arrowheads indicate amoeboid microglia colocalizing with strong MHCII signal (**C**_**6**_–**C**_**8**_). Arrowheads indicate ramified microglia colocalizing with low MHCII signal (**C**_**10**_–**C**_**12**_)

**Fig. 3:**
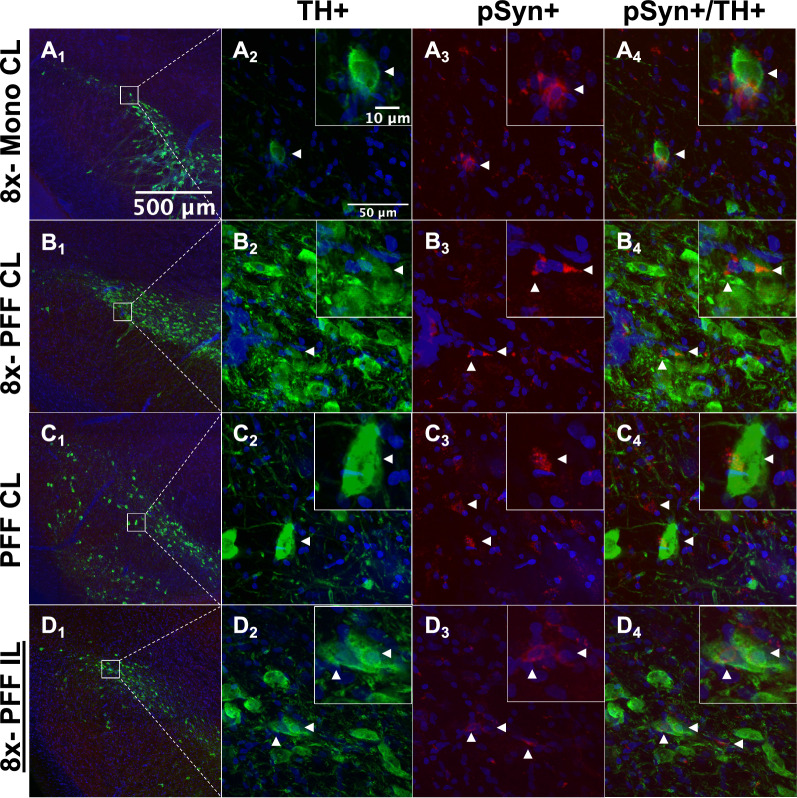
Rare false inclusions that meet 2 of the three criteria for authentic Lewy Body-like inclusions are present in or near dopaminergic neurons. 8× r-mTBI side contralateral to monomer or PFF injection stained positive for aggregated pSyn but the inclusions were partially or entirely extracellular. Side contralateral to PFF injection without 8× r-mTBI also displayed rare false inclusions (**A**_**1**_–**C**_**4**_). Authentic Lewy body-like inclusions have a perinuclear spiral contained within the TH+ cell and are near other cells with either authentic Lewy body-like inclusions or Lewy neurites (**D**_**1**_–**D**_**4**_). White arrowheads indicate TH+ neuron of interest with pSyn channel off (**A**_**2**_, **B**_**2**_, **C**_**2**_, and **D**_**2**_). Arrowheads indicate false inclusions (**A**_**3**_–**A**_**4**_, **B**_**3**_–**B**_**4**_, **C**_**3**_–**C**_**4**_) and authentic inclusions in (**D**_**3**_–**D**_**4**_)

**Fig. 4:**
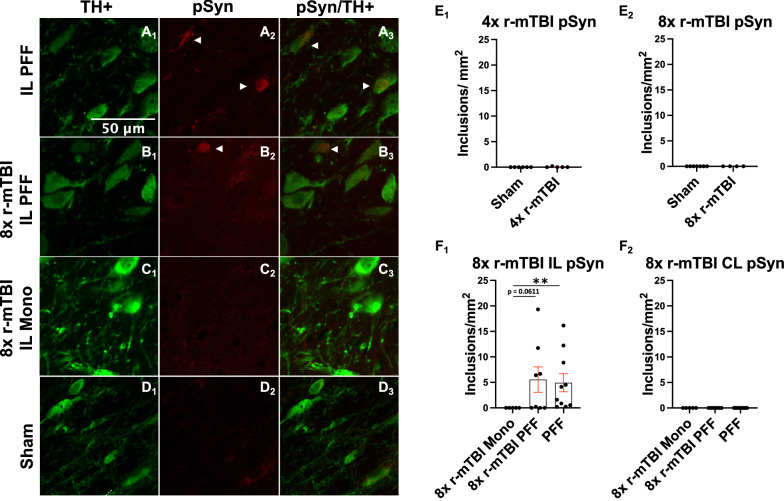
Quantification of Lewy body-like phospho-Serine-129 positive αSyn pathology in the SNpc. Representative images of αSyn pathology in dopaminergic neurons present in the SNpc ipsilateral to PFF injection (**A**_**1**_–**B**_**3**_). αSyn pathology is not present in monomeric αSyn injected into the SNpc and sham rat SNpc (**C**_**1**_–**D**_**3**_). Quantification of αSyn aggregates in rats that underwent either 4 or 8 r-mTBIs compared to their controls (**E**_**1**_–**E**_**2**_). αSyn pathology is present in the SNpc ipsilateral to the PFF injection (**F**_**1**_) and not on the contralateral side (**F**_**2**_). White arrowheads indicate authentic, Lewy Body-like inclusions localized to TH+ dopaminergic neurons in the SNpc (**A**_**2**_–**A**_**3**_**, B**_**2**_–**B**_**3**_)

Injury severity was titrated on unshielded anesthetized adult male SD rats using metal rods 0–2.0 kg with 0.250 kg increments. 2.0 kg injury was instantly lethal, without causing a skull fracture (Fig. 1). 1.750 kg injury was the highest survivable single injury (1.75 kg released from 25 cm and calculated impact of 4.29 J). The animals showed no abnormal behavior in grooming, body mass or motor performance (Fig. 1C). On two separate occasions, a second injury of 1.75 kg caused death after 14-day interval from the first injury without skull fracture, and with immediate swelling in the area (Fig. 1B). Upon dissection of the carcass profound subdural bleeding was identified. 1.75 kg was therefore designated as a traumatic brain injury because it was a single survivable injury severity that could not be tolerated a second time. 1.5 kg released from 25 cm with calculated impact of 3.68 J was the highest tolerated repetitive injury. We therefore defined it as mild TBI (mTBI). 4 consecutive r-mTBIs did not produce pathology detectable by IHC but 8 injuries 14 days apart did produce encephalomalacia throughout the brain without motor or behavioral deficits (Fig. 1). These areas of brain thinning were infiltrated by MHCII expressing microglia and astroglial expansion (Fig. 1D1–D10). Areas of encephalomalacia were visible on the intact brain before sectioning (Fig. 1D11). To accommodate a battery of behavioral experiments interval typically 10 days was extended to 14 days. No differences were detected between groups in the open field (Fig. 1C). No differences were detectable in novel object and novel placement tests (Fig. 2S). Behavioral assays on subsequent cohorts that underwent intracranial injections were not performed. In general, there appeared to be more inflammation in brain sections from 8× r-mTBIs at these time points compared to shams who had inflammation seen even outside the immediate regions of encephalomalacia (Fig. 1S). s100Beta, an astrocytic protein previously reported in blood of patients [37, 38] and rats [39] after a TBI. Permeability of the blood brain barrier (BBB) was measured but not detected at 2 h, a timepoint previously reported to produce most BBB permeability rats after a blast TBI [36]. s100beta was not detected using western blot (Fig. 3S) or ELISA (Fig. 4S).

### Dopaminergic neuron loss caused by injection of PFFs was not accelerated by r-mTBI

We have previously shown that at 3 months post PFF injection there was a ~ 20% DN loss ipsilateral to the injection accompanied by the spread of αSyn pathology [31]. Injection of recombinant αSyn fibrils caused progressive neurodegeneration on the side ipsilateral to the injection (Fig. 2). Fibrilized αSyn induced pathology was not accelerated by 8× r-mTBIs (Fig. 2). Combination of 8× r-mTBIs with monomeric vehicle surgery did not cause additional dopaminergic neurodegeneration (Fig. 2). MHCII + iba1+ microglia were present in the nigra ipsilateral to all injected groups (Fig. 2C1–C12) most notably in 8× r-mTBI + PFF group (Fig. 2C6–C8). These results are summarized in (Table 2).

**Table 2:** Summary of r-mTBI and PFF induced PD like pathology in the SNpc

	Left				Right			
	pTau	aSyn	TH loss	Iba1 + MHCII	pTau	aSyn	TH loss	Iba1 + MHCII
8× r-mTBI + PFF	+++	0	0	+++	+++	+++	++	+++
PFF	+	0	0	0	+++	+++	++	++
8× r-mTBI + Mono	+++	0	0	+++	+++	0	0	+++
8× r-mTBI	+++	0	0	+++	+++	0	0	+++
4× r-mTBI	+	0	0	0	+	0	0	0
Sham	+	0	0	0	+	0	0	0

Relative abundance of pathology is listed in descending order of burden in the left and the right side of the brain. For rats that underwent intracranial injection, right side is ipsilateral to the injection

### False positive αSyn aggregates

Images are of rare events that met only 2 of the 3 αSyn inclusion criteria and were therefore excluded from total counts. Aggregates are completely or partially localized to a TH+ soma and are adjacent to the nucleus (Fig. 3A1–C4). False inclusions are not in the vicinity of authentic Lewy body-like or Lewy neurite-like authentic inclusions. The false inclusions have irregular and punctate shape and lack perinuclear spiral typical of authentic Lewy bodies (Fig. 3D1–D4).

### αSyn pathology caused by injection of PFFs into the SNpc was not accelerated by 8× r-mTBI

Phospho-Ser129 + αSyn inclusions were present only in ipsilateral nigra of PFF injected rats and r-mTBI did not increase the αSyn inclusion burden or produce it de novo (Fig. 4). 4× r-mTBI did not cause production of authentic LB-like inclusions (Fig. 4E1). Similarly, 8× r-mTBI did not cause formation of authentic LB-like inclusions (Fig. 4E2). Only the injection of PFFs caused a formation of LB-like αSyn inclusions ipsilateral to the injection (Fig. 4F1–F2), and this pathology was not accelerated by 8× r-mTBIs. Contralateral side of animals that received PFF injection and/or r-mTBI was free of αSyn inclusion pathology (Fig. 4F1–F2). Representative images of αSyn inclusions that are present in nigra ipsilateral to the PFF injection (Fig. 4A1–B3) and also absent in injection controls that also received r-mTBI or sham animals are shown (Fig. 4C1–D3).

### Hyperphosphorylated Tau aggregation in the SNpc was increased by PFF injection or by r-mTBI

**Fig. 5:**
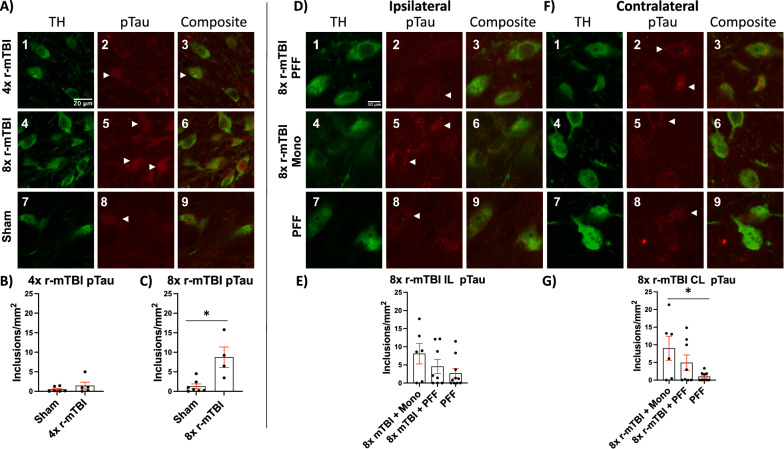
Quantification of pTau positive pathology in the SNpc. Representative images of 4× and 8× r-mTBI pTau pathology localized to TH+ dopaminergic neurons (**A**_**1**_–**A**_**9**_). Counts of pTau inclusions in TH+ neurons in shams and 4× r-mTBI rat SNpc (**B**). Counts of pTau inclusions in TH+ neurons in shams and 8× r-mTBI rat SNpc (**C**). Representative images of objects counted in monomer or PFF injected groups with and without 8× rmTBIs, ipsilateral to the injection (**D**_**1**_–**D**_**9**_). Counts of pTau inclusions in TH+ neurons in the SNpc ipsilateral to the injection of PFFs or monomers with and without 8× r-mTBI (**E**). Representative images of objects counted in monomer or PFF injected groups with and without 8× r-mTBIs, contralateral to the injection (**F**_**1**_–**F**_**9**_). Counts of pTau inclusions in TH+ neurons in the SNpc contralateral to the injection of PFFs or monomers with and without 8× r-mTBI (**G**). White arrowheads indicate pTau aggregate pathology localized to TH+ dopaminergic neuron that was counted

pTau positive aggregate burden localized to DN increased as a result of 8× r-mTBI or PFF injection. Representative images of objects counted in 4× and 8× r-mTBI groups (Fig. 5A1–A9). Few aggregates with no significant differences were found in sham animals and 4× r-mTBI (Fig. 5B). 8× r-mTBIs caused a significant increase in the pTau positive aggregates (Fig. 5C). Representative images of objects counted in monomer or PFF injected groups with and without 8× r-mTBIs, ipsilateral to the injection (Fig. 5D1–D9). No differences were detected between groups of rats that received injection and r-mTBI compared to PFF injection alone on the ipsilateral side. (Fig. 5E). Representative images of objects counted in monomer or PFF injected groups with and without 8× r-mTBIs, contralateral to the injection (Fig. 5F1–F9). 8× r-mTBI caused an increase in pTau on the contralateral side (Fig. 5G). These results are summarized in (Table 2).

### pTau aggregate morphology and intensity differ between groups

**Fig. 6:**
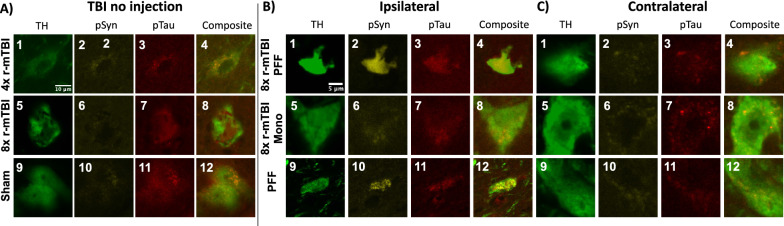
Qualitative comparison of pTau positive inclusions localized to TH+ dopaminergic neurons. Representative images of αSyn and pTau pathology in dopaminergic neurons present in the SNpc of animals that underwent 8× r-mTBI +/− PFF or monomer injection. 4× r-mTBI pTau (**A**_**1**_–**A**_**4**_). 8× r-mTBI (**A**_**5**_–**A**_**8**_). Sham control (**A**_**9**_–**A**_**12**_). 8× r-mTBI + PFF ipsilateral (**B**_**1**_–**B**_**4**_), contralateral (**C**_**1**_–**C**_**4**_). 8× r-mTBI + Mono ipsilateral **B**_**5**_–**B**_**8**_), contralateral (**C**_**5**_–**C**_**8**_). PFF injected no TBI, ipsilateral (**B**_**9**_–**B**_**12**_), contralateral (**C**_**9**_–**C**_**12**_)

Representative images of 8× r-mTBI and PFF injection that produced different pTau aggregate formation in DNs of the SNpc. αSyn inclusions were only visible on the side ipsilateral to the PFF injection (Fig. 6). pTau aggregates present in the sham group were least intense and were segmented or punctate in appearance (Fig. 6A11–A12). 8× r-mTBI produced pTau aggregates that were solid and more intense (Fig. 6A7-A8) than 4× r-mTBI (Fig. 6A3–A4) or sham (Fig. 6A11–A12). 8× r-mTBI + PFF produced solid intense staining inclusions in the DNs on the injection ipsilateral side that colocalized with αSyn aggregates (Fig. 6B1–B4). Punctate pTau aggregates were present in 8× r-mTBI + PFF contralateral side (Fig. 6C1–C4). 8× r-mTBI + Monomer produced punctate pTau aggregates on the ipsilateral and contralateral sides (Fig. 6B5–B8, C5–C8). PFF injection without TBI produced spiral αSyn inclusions (Fig. 6B9–B12) that colocalized with intense punctate pTau staining, while the contralateral side displayed some punctate pTau at a low intensity (Fig. 6C9–C12).

## Discussion

### Modeling r-mTBI

Numerous rodent TBI models were developed over the years to help answer specific questions about TBI pathology [21, 40]. Weight drop instruments, initially developed by Maramarou, were most widely adopted for a variety of applications, including mild repetitive injuries in mice [41]. Rats, however, have more complex motor behaviors which would make their application to the study of movement disorders like PD preferable [42]. Rat genetics [43] and pharmacokinetics [44] are also closer to humans compared to mice making them a better animal model candidate for study of gene-environment interactions and protein expression levels. With this in mind, we developed a weight drop model to test whether r-mTBI caused de novo or accelerated existing αSyn pathology central to PD.

No two human TBIs are the same, and they are unlikely to occur in the same focal area, therefore we sought to create a preclinical model of brain injury that reflects the variable nature of human head trauma. This preclinical model of TBI was designed to simulate the most common causes of head trauma reported in the clinic which include car accidents, falls or a strike to the head [45]. In these types of injuries, the surface of an object connects with large portion of the calvarium, rather than a single focal point. The head is typically not immobilized during human TBIs, and a strike often causes rotational acceleration which in and of itself can produce damage to the brain [46]. For the vast majority of individuals, symptoms associated with a mTBI, which include nausea, headaches, and blurred vision resolve within 7–10 days [47–49]. We reasoned that a repeated injury was less likely to occur during this convalescent period because the individual may be too ill to engage in the same type of behavior that led to the TBI or may follow physician’s advice to abstain from such activities. Therefore, we set our injury interval to 10 or 14 days if behavioral experiments are being performed. However, it is important to recognize that if the injury interval was shorter that the pathology and behavioral deficits may be more apparent. Recently, Buchele et al. used rods of 2.5 kg placed 25 cm above the rat to achieve a TBI on a shielded rat cranium [29]. Similarly, to establish parameters for unshielded injury we performed a titration 0–2.0 kg with 0.250 kg increments from 25 cm height. 2.0 kg injury was instantly lethal, and 1.75 kg injury was survivable only once, causing instant death upon second injury 10–14 days after the initial injury. No motor or behavioral deficits were observed after single 1.75 kg injury. This level of injury could be survived only once, and it was designated as a severe traumatic brain injury. A mild traumatic injury was defined as the highest tolerated repetitive injury, which was 1.5 kg. In these injuries the rat head was unrestrained on a sponge to allow for rotational acceleration upon impact. Next, we wanted to determine how many mild injuries were necessary to cause detectable pathology similar to what is seen on neuroimaging in patients with a history of TBI where regional encephalomalacia or brain thinning can be detected [30]. Published focal injury models and mTBI models requiring preparatory surgery ranged from 2 to 10 injuries, some occurring seconds apart while others are weeks apart [50–53]. Initial experiments were performed with 4 injuries separated 14 days apart to allow for full recovery and to test for residual long lasting effects in the second week after injury. Surprisingly, no pathology and no behavioral deficits were detected after 4 injuries. The number of injuries were subsequently doubled to 8 injuries which were sufficient to cause areas of encephalomalacia, that are demarcated by increased microglia (Iba1+) expressing MHCII a marker not expressed in the brain under non pathological conditions. Additionally, astrocyte expansion in regions of encephalomalacia (GFAP+) was observed (Fig. 1). These animals displayed normal righting reflex after each injury, no deficits in behavior, grooming, swelling at the site of injury, and maintained normal weight (data not shown). Therefore, 8 injuries using 1.5 kg rods released from 25 cm height were used to determine if r-mTBI accelerated existing PD pathology in subsequent experiments.

### r-mTBI does not cause or accelerate fibrilized αSyn induced PD-like pathology in the SNpc at the 3 month endpoint

Robust PD-like neurodegeneration accompanied by αSyn inclusion deposition in the surviving neurons was previously reported in rats at 3 months following an intracranial injection of PFFs [26, 54, 55]. 8× r-mTBIs were found to cause pathology throughout the brain most notably in the cortex and to a lesser extent midbrain without detectable behavioral deficits (Fig. 1). Due to lack of detectable behavioral deficits after 8× r-mTBI, behavioral assays were not performed in subsequent cohort that underwent injection of fibrilized or monomeric control αSyn. However, behavioral deficits should be revisited at a later timepoint (e.g. 6 months post PFF injection) due to progression of both TBI and PD related pathologies. Horizontal ladder test may help detect PD related deficits that were not apparent in open field test performed at the 3-month endpoint. 8× r-mTBIs did not cause formation of pathology characteristic of PD such as aggregation of αSyn into characteristic Lewy bodies and Lewy neurites and DN loss in the SNpc (Fig. 4). Moreover, 8× r-mTBIs did not accelerate PD-like pathology caused by injection of recombinant αSyn fibrils (Figs. 2, 4). We therefore conclude that r-mTBIs do not cause or accelerate αSyn pathology at 3 months post fibrilized αSyn injection. While αSyn pathology in the nigra was not accelerated, other aspects of PD pathology may have been affected such as faster spread of inclusions from dopaminergic neurons to medium spiny neurons previously reported in fibril models [54]. r-mTBIs may increase expression and spread of other proteins that may indirectly affect abundance and exacerbate the burden of αSyn pathology. αSyn pathology may be slow to develop without the aid of fibrilized αSyn injection and later timepoints may show development of αSyn pathology. Axonal projections from DNs in the SNpc reach considerable distance, and therefore, they may be more susceptible to repetitive diffuse axonal shearing forces thereby severing or compromising connection between nigra and the striatum. Interruption of nigrostriatal signaling pathway would in turn worsen PD pathology by disrupting proper dopamine signaling without necessarily causing buildup of αSyn inclusions. Over time, damage to long DN projections may cause dieback of DN soma in the SNpc. Damage to dopamine projections into the striatum caused by striatal injection of neurotoxin, 6 hydroxydopamine, caused progressive axonal degeneration that eventually resulted in DN cell death in the SNpc [56]. It is also possible that the injury severe enough to cause PD pathology in nigra, in rats as in humans, by either nucleating αSyn pathology or by causing aggressive loss of DNs is lethal in most cases [57].

### pTau aggregation in the SNpc of rats after r-mTBI with and without preexisting PD-like pathology

Tau is soluble microtubule associated protein, whose main function is the stabilization of microtubule assembly networks. The exact role of Tau in TBI and PD remains incompletely understood. Hyperphosphorylated Tau (pTau) can cause loss of function resulting from destabilized microtubule network and deposition of toxic pTau aggregates [58], and has been shown to accelerate aggregation of αSyn in vitro [59, 60], however, presence or absence of endogenous pTau was found not to impact aggregation of αSyn in vitro and in vivo even though Tau colocalized with αSyn aggregates [61]. pTau has been shown to localize to Lewy Bodies in idiopathic PD [62], and has been linked to consequence of TBI in humans and mice [63, 64]. The role of pTau in nigra, following a TBI and during development of PD, has not been studied in the SNpc and was therefore of great interest.

Increased pTau aggregation was found in rats that either underwent r-mTBI or a PFF injection to cause PD-like pathology (Fig. 5). These findings were not unexpected because pTau aggregates of various types were found to colocalize with Lewy bodies in human brains [62]. The degree of accumulation of pTau is similar in rats that underwent a 8× r-mTBI (Fig. 5C), regardless of whether or not they also underwent an injection of either PFFs or monomeric αSyn (Fig. 5E, G). Increased pTau aggregation in monomer injected was likely due to 8× r-mTBI and not monomer injection alone, which would be in line with previous studies that showed no pathology induced by injection of monomeric αSyn [26, 31, 54]. Regions contralateral to injection show that pTau aggregation burden is highest in rats that underwent r-mTBI compared to PFF injected alone (Fig. 5G). It would be of interest to determine if the presence of pTau before nucleation of αSyn pathology, by injection of fibrils, can promote faster αSyn aggregation. Identification of pTau aggregates in dopaminergic neurons of the SNpc after r-mTBIs may provide an opportunity to slow down the progression of the diseases using pTau specific therapeutics originally developed for treatment of Alzheimer’s disease [65, 66].

### pTau aggregation resulting from r-mTBI may nucleate αSyn pathology directly by cross-seeding or indirectly by perturbing proteosome/lysosomal pathways

Cross-seeding or corrupt templating has been shown to occur between αSyn and Tau as well as other proteins [67, 68]. Increased pTau aggregation resulting from repetitive mild brain injuries may, in time, directly nucleate aggregation of αSyn serving as a corrupting template. It is also possible that cellular sequestration of pTau and αSyn occurs through similar mechanisms and both are simply deposited in the same area within the cell. In this case, pTau aggregation can help create an environment in which spontaneous αSyn aggregation is more likely. Apparent ultrastructural differences between pTau aggregates caused by PFF injection and those caused by PFF + r-mTBI and r-mTBI were observed. pTau aggregates colocalized with PFF induced αSyn aggregates appear to be punctate (Fig. 6B9–B12) while those induced by 8× r-mTBI (Fig. 6A6–A8) or PFF + 8× r-mTBI are typically more solid (Fig. 6B1–B4). Interestingly, pTau aggregates on the contralateral side to injection of either PFF or monomer produced segmented or punctate pTau (Fig. 6C), while 8× r-mTBI without injection produced typically solid inclusions (Fig. 6A7–A8). Inflammation, likely caused by intracranial injection surgery may have had some influence on segmentation of aggregates because 8× r-mTBI without injection produced more solid aggregates. These apparent ultrastructural differences merit further research to determine toxicity or a maturation state of the aggregate. This could be determined by experiments with a later timepoint, e.g. 6 months. Acetylation of Tau was found to occur in the early stages of 3 postmortem Alzheimer’s and 3 postmortem CTE brains [69]. These diseases are known to develop pTau pathology at later stages, and acetylation, particularly at the lysine 280 residue may be an important precursor to pTau hyperphosphorylation [69]. Acetylation may also play a role in pTau aggregation found in PD. pTau aggregates with different toxicity and biochemical profiles were identified in mice that underwent single or repetitive TBI suggesting that different types of injuries may produce different type of pTau species [64]. Different types of pTau aggregates may have different ability nucleate pathology specific to PD. Phosphorylated TDP-43 is known to co-occur with pTau pathology in TBI and may be involved in cross seeding between pTau and αSyn. TDP-43 pathology is more common in human brains after r-mTBI than single severe TBI [70], and warrant additional attention in future studies using our rat model.

### Limitations

This TBI study was limited to male rats because most TBIs are reported in men who are at double the risk for PD [13, 71]. Different outcomes have been reported in women for PD and TBI and future studies designed specifically to understand sex differences are warranted [72, 73]. Unexpected mortalities required an increase in group size from n = 4 to n = 8. For subsequent cohorts that underwent an injection of either PD pathology causing fibrils or monomeric control, the n was increased to 10 in anticipation of higher mortality due to combined insults. Interestingly, there was significantly lower mortality from r-mTBI in animals that underwent injections with only 2 losses in 8× r-mTBI and fibril injected and 3 in monomeric vehicle injected controls that also received an 8× r-mTBIs. Variation in rat mortality may be because they were from separate cohorts of rats and cohort difference may have been a factor. These findings underscore our use of controls and test animals to avoid artificial stratification.

## Conclusions and future direction

Our results indicate that after at least 8 repetitive mild TBIs, chronic brain pathology is produced in rats reminiscent of neuroimaging findings in patients with a history of TBI. These injuries consist of encephalomalacia, ventricular enlargement, astrocyte expansion and microgliosis in the absence of motor deficits. We also found that repetitive mild TBIs caused an increased pTau deposition in DNs of the SNpc without altering preexisting Parkinson’s-like αSyn pathology burden. Role of pTau in the SNpc is an underexplored area which deserves additional attention. It would be of interest to determine if the presence of both pTau and αSyn agregates within dopaminergic neurons accelerates their demise by overburdening proteosome/lysosome degradation pathways at later timepoints. Additional fundamental questions about the relationship between r-mTBI induced pTau aggregation remain unanswered. For instance, whether nucleation of αSyn pathology after r-mTBI (that induced pTau aggregation) results in more aggressive spread of αSyn pathology induced by injection of recombinant fibrils.

## Supplementary Information

**Additional file 1. Figure 1S. 8x r-mTBI and age matched sham control brain sections stained for microglia iba1+ (green) and MHCII (red) and scanned using Li-COR odyssey. ** Sham striatum **1,5,9** and sham nigra **3,7,11** containing coronal sections show low iba+ and MHCII signal. 8x r-mTBI striatum **2,6,10** and 8x r-mTBI nigra **4,8,12** show microglial infiltration of injured brain areas with colocalization of iba1 and MHCII signal.

**Additional file 2. Figure 2S. Novel object recognition and placement were performed to test if r-mTBI causes learning and memory deficits in rats.** No differences detected after each of the 8 planned injuries in novel object recognition **A** or novel object placement **B**.

**Additional file 3. Figure 3S ELISA measure of blood serum S100beta as an indicator of the blood brain barrier integrity.** S100beta was undetectable in the rat blood serum two hours after either mild or severe TBI **A**. S100beta standard curve was generated with the protein provided in the kit **B**.

**Additional file 4. Figure 2S. Figure 4S. S100beta serum levels were measured with the 3 most frequently cited commercially available antibodies using western blot approaches.** S100beta was detected only in the brain homogenate with two antibodies **A,B** while the third antibody did not detect S100beta (**C**). White arrow indicates location of the faint 10 kDa band in brain homogenate controls.

**Additional file 5. Figure 5S. Dynamic light scattering of sonicated PFFs prior to injection.** Water bath sonication of PFF prep produced majority of fibril fragments with a diameter of 30 nm or less.

**Additional file 6. Figure 6S. TH+ neurons, pTau inclusions and αSyn inclusion were counted using image J.** TH staining was used identify SNpc and draw region of interest (ROI). αSyn and pTau aggregates localized to TH+ cell bodies were manually counted and normalized to the area of the ROI. TH+ dopaminergic neurons containing αSyn pathology **A**. TH + neurons in SNpc containing pTau pathology **B**. TH+ SNpc neurons of sham control rats containing no aggregates **C**. White arrowheads indicate localization of pTau or αSyn inclusions as they appeared in the images used to count inclusions localized to TH+ SNpc neurons.

## Data Availability

The datasets used and/or analyzed during the current study available from the corresponding author on reasonable request. The data set will also be available through Open Data Commons for Traumatic Brain Injury website.

## Abbreviations

DN: Dopaminergic neuron
GFAP: Glial fibrillary acidic protein
IACUC: Institutional Animal Care and Use Committee
IBA1: Ionized calcium binding adaptor molecule 1
LB: Lewy body
LN: Lewy neurite
LPS: Lipopolysaccharide
MHCII: Major histocompatibility complex II
Mono: Monomeric alpha synuclein
mTBI: Mild TBI
PD: Parkinson’s disease
PFF: Pre formed alpha synuclein fibrils
pTau: Hyper phosphorylated Tau
r-mTBI: Repetitive mild TBI
SD: Sprague Dawley
SNpc: Substantia nigra pars compacta
TBI: Traumatic brain injury
TH: Tyrosine hydroxylase
αSyn: Alpha synuclein
