# Supplementary material for: Repetitive mild TBI causes pTau aggregation in nigra without altering preexisting fibril induced Parkinson’s-like pathology burden
Source: Acta Neuropathol Commun. 2022 Nov 26;10:170. doi: 10.1186/s40478-022-01475-9 (PMC9701434; doi:10.1186/s40478-022-01475-9)
Supplement: Supplementary file 1 — Additional file 1. Figure 1S. 8x r-mTBI and age matched sham control brain sections stained for microglia iba1+ (green) and MHCII (red) and scanned using Li-COR odyssey. Sham striatum 1,5,9 and sham nigra 3,7,11 containing coronal sections show low iba+ and MHCII signal. 8x r-mTBI striatum 2,6,10 and 8x r-mTBI nigra 4,8,12 show microglial infiltration of injured brain areas with colocalization of iba1 and MHCII signal. [file 40478_2022_1475_MOESM1_ESM.pdf]

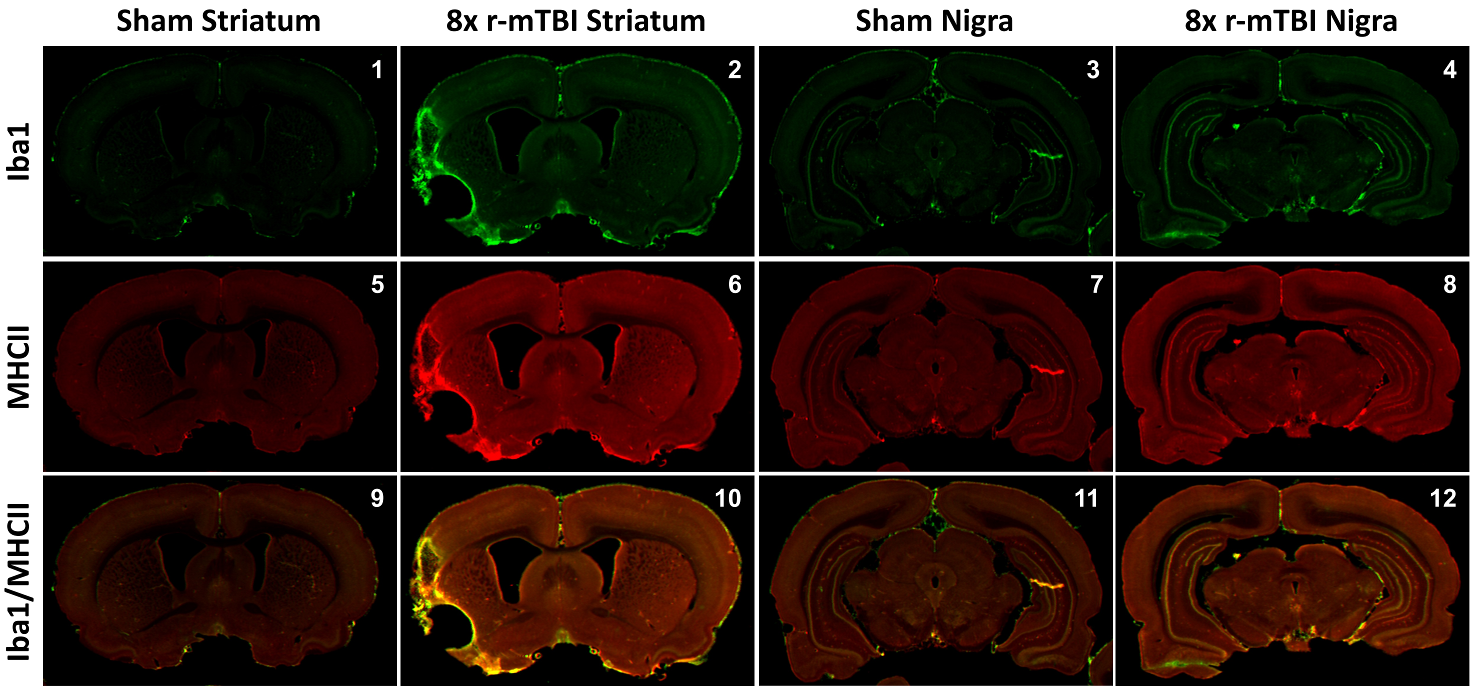

Figure 1S. 8x r-mTBI and age matched sham control brain sections stained for microglia iba1+ (green) and MHCII (red) and scanned using Li-COR odyssey. Sham striatum **1,5,9** and sham nigra **3,7,11** containing coronal sections show low iba+ and MHCII signal. 8x r-mTBI striatum **2,6,10** and 8x r-mTBI nigra **4,8,12** show microglial infiltration of injured brain areas with colocalization of iba1 and MHCII signal**.**
