# Supplementary material for: Repetitive mild TBI causes pTau aggregation in nigra without altering preexisting fibril induced Parkinson’s-like pathology burden
Source: Acta Neuropathol Commun. 2022 Nov 26;10:170. doi: 10.1186/s40478-022-01475-9 (PMC9701434; doi:10.1186/s40478-022-01475-9)
Supplement: Supplementary file 2 — Additional file 2. Figure 2S. Novel object recognition and placement were performed to test if r-mTBI causes learning and memory deficits in rats. No differences detected after each of the 8 planned injuries in novel object recognition A or novel object placement B. [file 40478_2022_1475_MOESM2_ESM.pdf]

A)

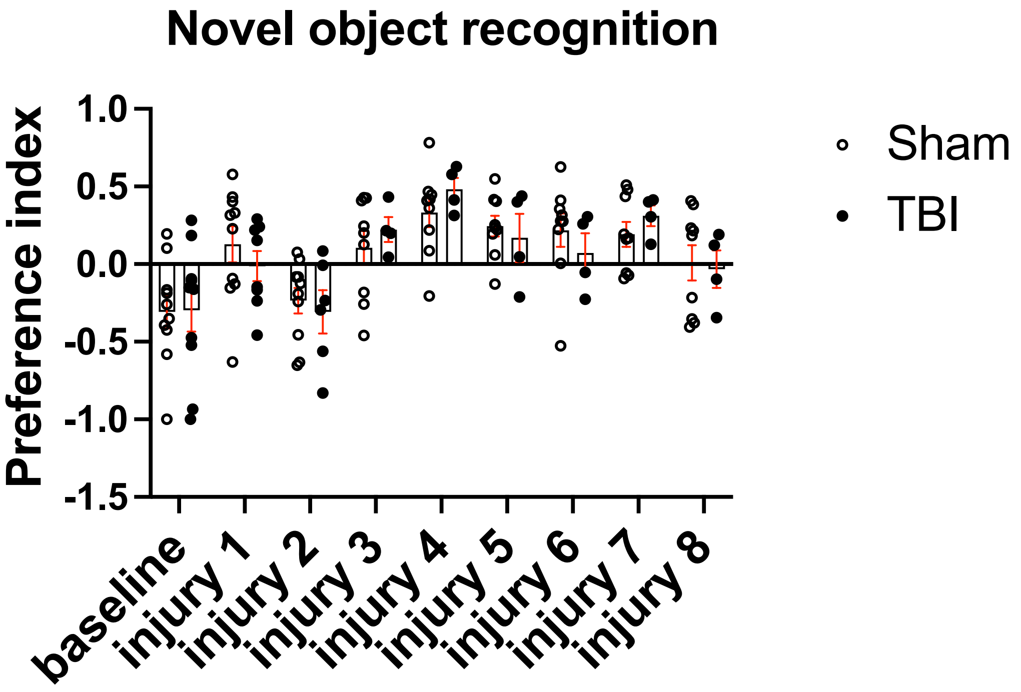

B)

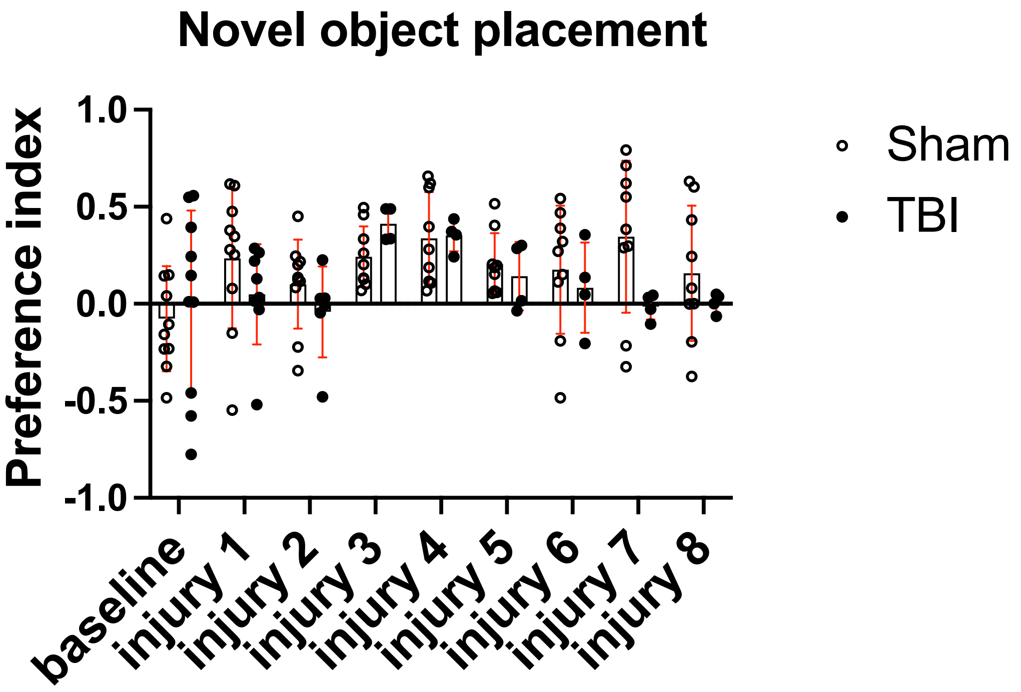

Figure 2S. Novel object recognition and placement were performed to test if r-mTBI causes learning and memory deficits in rats. No differences detected after each of the 8 planned injuries in novel object recognition **A** or novel object placement **B.**
