# Supplementary material for: Repetitive mild TBI causes pTau aggregation in nigra without altering preexisting fibril induced Parkinson’s-like pathology burden
Source: Acta Neuropathol Commun. 2022 Nov 26;10:170. doi: 10.1186/s40478-022-01475-9 (PMC9701434; doi:10.1186/s40478-022-01475-9)
Supplement: Supplementary file 3 — Additional file 3. Figure 3S ELISA measure of blood serum S100beta as an indicator of the blood brain barrier integrity. S100beta was undetectable in the rat blood serum two hours after either mild or severe TBI A. S100beta standard curve was generated with the protein provided in the kit B. [file 40478_2022_1475_MOESM3_ESM.pdf]

A)

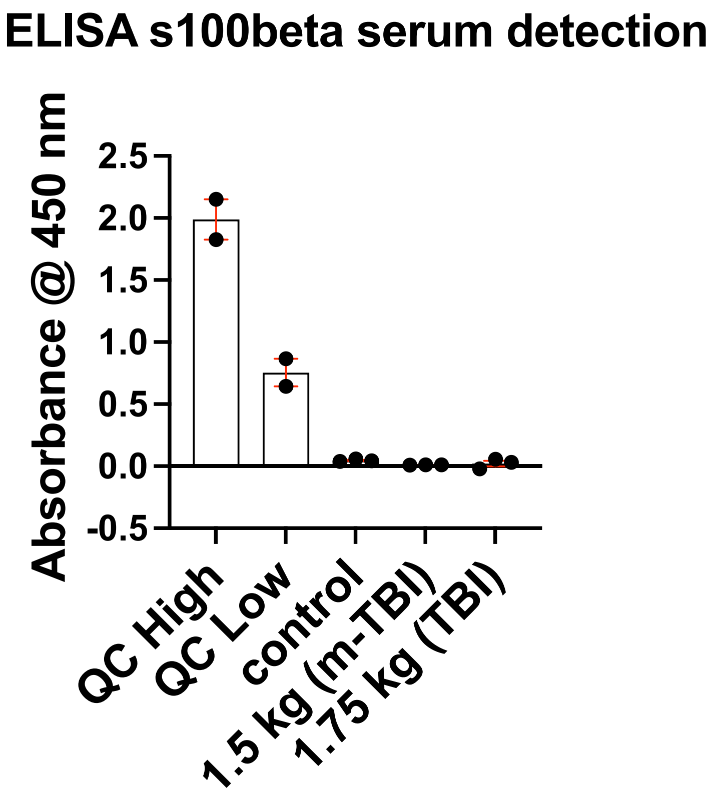

B)

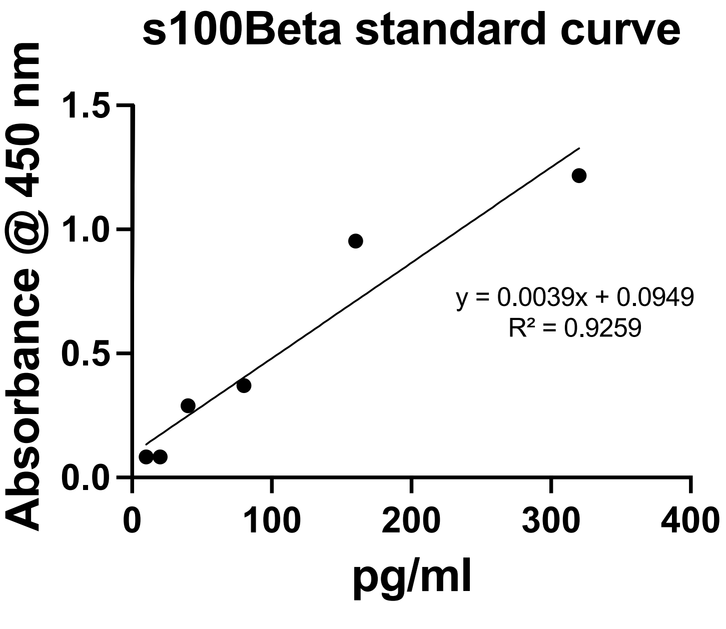

Figure 3S ELISA measure of blood serum S100beta as an indicator of the blood brain barrier integrity. S100beta was undetectable in the rat blood serum two hours after either mild or severe TBI **A**. S100beta standard curve was generated with the protein provided in the kit **B**.
