# Supplementary material for: Repetitive mild TBI causes pTau aggregation in nigra without altering preexisting fibril induced Parkinson’s-like pathology burden
Source: Acta Neuropathol Commun. 2022 Nov 26;10:170. doi: 10.1186/s40478-022-01475-9 (PMC9701434; doi:10.1186/s40478-022-01475-9)
Supplement: Supplementary file 4 — Additional file 4. Figure 2S. Figure 4S. S100beta serum levels were measured with the 3 most frequently cited commercially available antibodies using western blot approaches. S100beta was detected only in the brain homogenate with two antibodies A,B while the third antibody did not detect S100beta (C). White arrow indicates location of the faint 10 kDa band in brain homogenate controls. [file 40478_2022_1475_MOESM4_ESM.pdf]

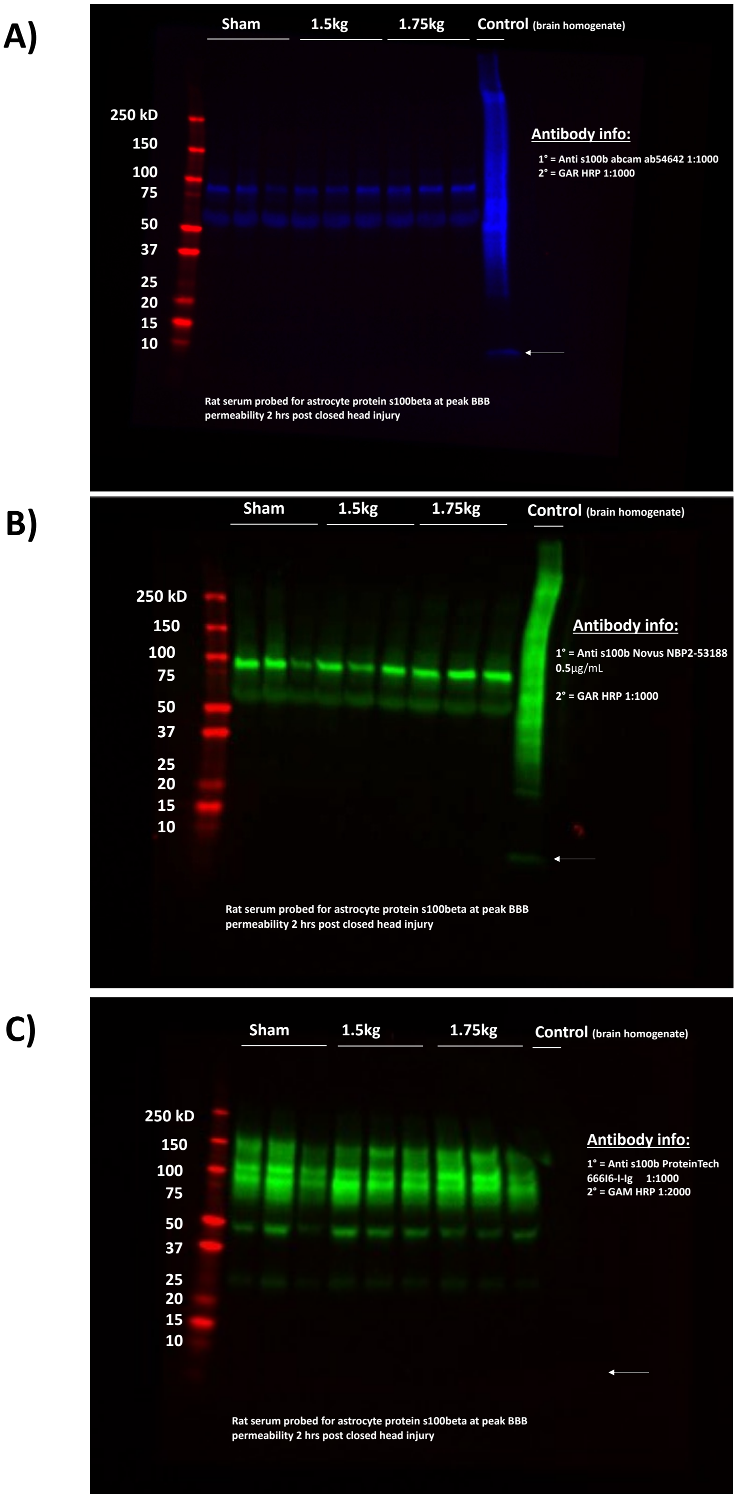

Figure 4S. S100beta serum levels were measured with the 3 most frequently cited commercially available antibodies using western blot approaches. S100beta was detected only in the brain homogenate with two antibodies **A,B** while the third antibody did not detect S100beta **(C).** White arrow indicates location of the faint 10 kDa band in brain homogenate controls.
