# Supplementary material for: Repetitive mild TBI causes pTau aggregation in nigra without altering preexisting fibril induced Parkinson’s-like pathology burden
Source: Acta Neuropathol Commun. 2022 Nov 26;10:170. doi: 10.1186/s40478-022-01475-9 (PMC9701434; doi:10.1186/s40478-022-01475-9)
Supplement: Supplementary file 5 — Additional file 5. Figure 5S. Dynamic light scattering of sonicated PFFs prior to injection. Water bath sonication of PFF prep produced majority of fibril fragments with a diameter of 30 nm or less. [file 40478_2022_1475_MOESM5_ESM.pdf]

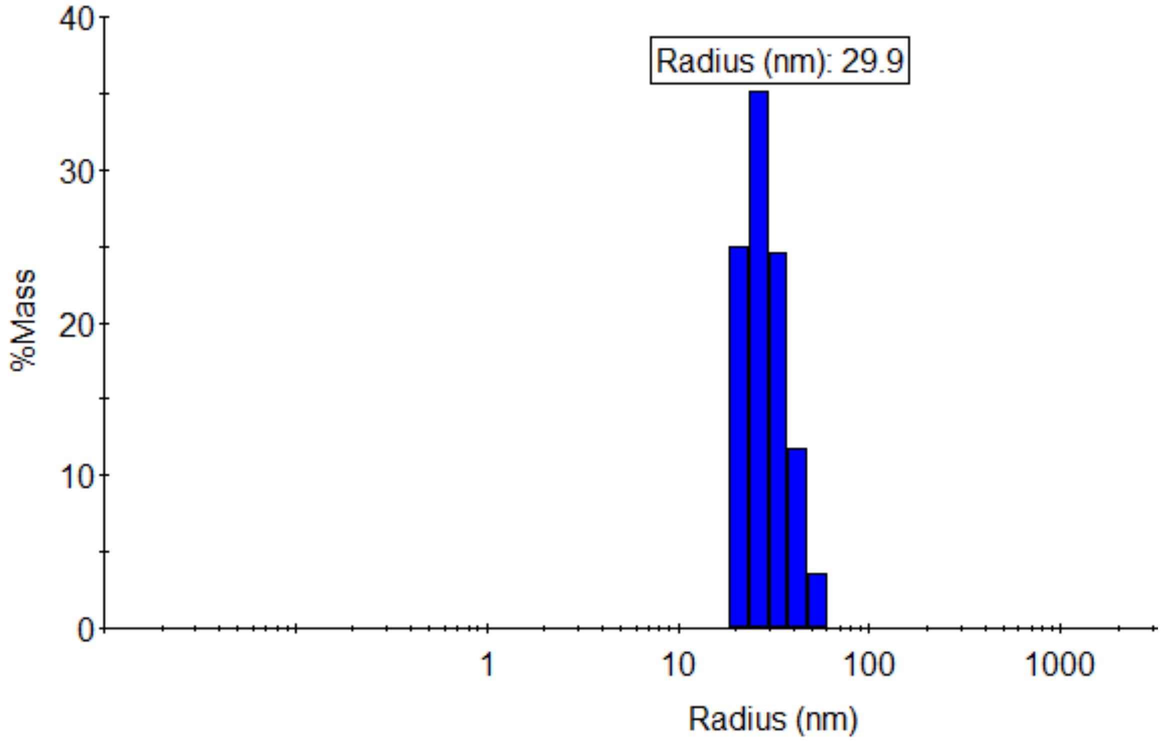

Figure 5S. Dynamic light scattering of sonicated PFFs prior to injection. Water bath sonication of PFF prep produced majority of fibril fragments with a diameter of 30 nm or less.
