# Supplementary material for: Repetitive mild TBI causes pTau aggregation in nigra without altering preexisting fibril induced Parkinson’s-like pathology burden
Source: Acta Neuropathol Commun. 2022 Nov 26;10:170. doi: 10.1186/s40478-022-01475-9 (PMC9701434; doi:10.1186/s40478-022-01475-9)
Supplement: Supplementary file 6 — Additional file 6. Figure 6S. TH+ neurons, pTau inclusions and αSyn inclusion were counted using image J. TH staining was used identify SNpc and draw region of interest (ROI). αSyn and pTau aggregates localized to TH+ cell bodies were manually counted and normalized to the area of the ROI. TH+ dopaminergic neurons containing αSyn pathology A. TH + neurons in SNpc containing pTau pathology B. TH+ SNpc neurons of sham control rats containing no aggregates C. White arrowheads indicate localization of pTau or αSyn inclusions as they appeared in the images used to count inclusions localized to TH+ SNpc neurons. [file 40478_2022_1475_MOESM6_ESM.pdf]

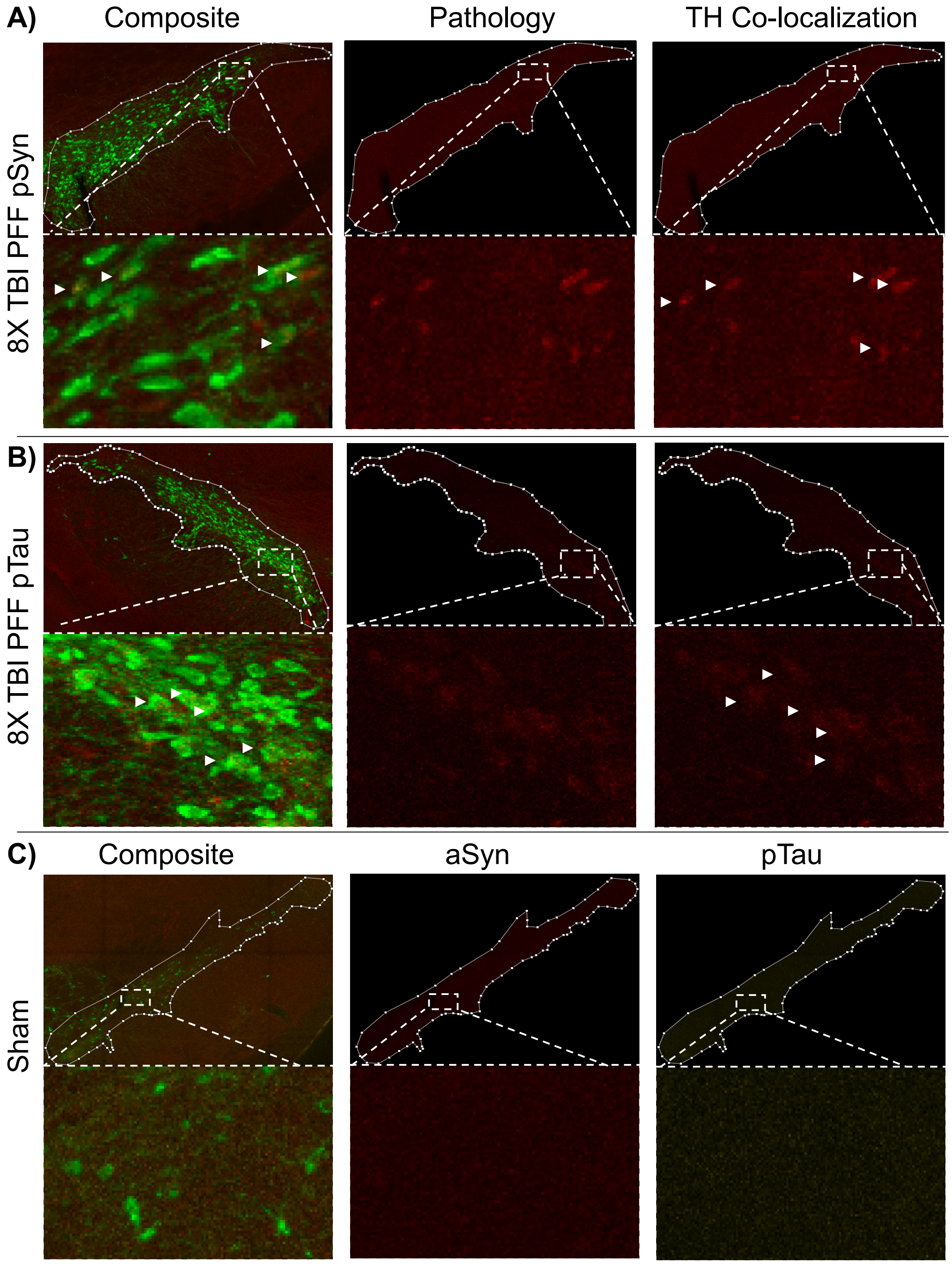

Figure 6S. TH+ neurons, pTau inclusions and αSyn inclusion were counted using image J. TH staining was used identify SNpc and draw region of interest (ROI). αSyn and pTau aggregates localized to TH+ cell bodies were manually counted and normalized to the area of the ROI. TH+ dopaminergic neurons containing αSyn pathology **A.** TH + neurons in SNpc containing pTau pathology **B.** TH+ SNpc neurons of sham control rats containing no aggregates **C.** White arrowheads indicate localization of pTau or αSyn inclusions as they appeared in the images used to count inclusions localized to TH+ SNpc neurons.
